# Suicide Mortality in Comparison to Traffic Accidents and Homicides as Causes of Unnatural Death. An Analysis of 14,441 Cases in Germany in the Year 2010

**DOI:** 10.3390/ijerph9030924

**Published:** 2012-03-15

**Authors:** Karoline Lukaschek, Natalia Erazo, Jens Baumert, Karl-Heinz Ladwig

**Affiliations:** 1 Institute of Epidemiology II, Helmholtz Zentrum München, German Research Centre for Environmental Health, Ingolstädter Landstrasse 1, 85764 Neuherberg, Germany; Email: baumert@helmholtz-muenchen.de (J.B.); ladwig@helmholtz-muenchen.de (K.-H.L.); 2 Department for Psychosomatic Medicine and Psychotherapy, Technische Universität München, Langerstrasse 3, 81675 Munich, Germany; Email: n.erazo@lrz.tu-muenchen.de

**Keywords:** unnatural death, suicide, traffic accidents, homicide, years of life lost, Germany, suicide prevention

## Abstract

*Aim*: To assess suicide mortality in comparison to traffic accidents and homicide deaths in Germany in the year 2010 and to compare years of life lost (YLL) due to these unnatural deaths. *Methods*: Mortality data were provided by the Federal Statistical Office giving death rates (related to 100,000 inhabitants) and proportions (related to 100 deaths of individuals) for suicide, traffic accidents and homicide as well as YLL data. *Results*: A total of 14,441 unnatural deaths (suicide, traffic accidents, homicide) were reported in 2010 in Germany. Of those, 10,021 subjects (69.4%) committed suicide, 3,942 (27.3%) died in traffic accidents, 478 (3.3%) were murdered. Suicide death rates were by far the highest, with rates for men (18.6) three times higher than for women (6.1). For both sexes, suicide rates increased with age, whereas suicide as a proportion of all causes of death was higher in younger age groups. In both sexes, suicide was the leading cause of YLL (men: 314 YLL, women: 90 YLL). *Conclusions*: Suicide is the leading cause of unnatural death and YLL. The sex- and age- specific patterns in suicide mortality call for different action plans to target high risk groups.

## 1. Introduction

Unnatural deaths are caused by external factors which include death due to intentional injury such as homicide or suicide, and death caused by unintentional injury in an accidental manner, such as traffic accidents. Unnatural deaths are of major concern for public health care and politics. In order to assess population health, health care providers can look at deaths that should not occur in the presence of effective and timely health care, so-called “avoidable” mortality [1]. The concept of “avoidable mortality” has provided a means to examine the quality of health care in a health care system and to identify topics for further in-depth investigation [2]. Avoidable mortality was intended as an indicator of potential weaknesses in health care and public health. It might be an appropriate tool to draw attention to problems that might otherwise have been missed.

The three main causes of unnatural death, suicide, traffic accidents and homicide, are dealt with very differently in public: Traffic safety issues and the prevention of traffic accidents are frequently discussed by politicians and interest groups. Homicides are omnipresent in the media and public, so that the actual numbers are fairly overestimated by the populace [3]. Suicide and suicide prevention strategies, on the other hand, receive less attraction by health politics, partly due to the fact that the topic of suicide can still be seen as a taboo in Western societies. The true proportions of the three different unnatural causes of death are not well-established in the society. Therefore, the major aim of this study was to fill this gap by assessing the actual burden of suicide mortality in comparison to traffic accidents and homicide deaths in a Western society using population based mortality data from Germany in the year 2010. 

## 2. Material and Methods

Mortality and population data provided by the Federal Statistical Office of Germany [4] were used to calculate rates and proportions of unnatural deaths (suicide, traffic accidents, homicides). Event rates were estimated for 100,000 inhabitants, event proportions for 100 deaths. Event rates give the amount of unnatural deaths in specific subgroups whereas event proportions show the amount of unnatural deaths in all death causes. The population base consisted of all residents of Germany who died from suicide, traffic accidents, or homicide in the year 2010. Death by suicide was defined as “intentional self-harm” according to the ICD-10 categories X60 to X84; death due to homicide was defined according to the ICD-10 categories X85 to Y09; traffic accident deaths were defined according to the ICD-10 categories V01 to V99. 

Years of Life Lost (YLL) were obtained by the Federal Statistical Office in Germany [4]. YLL represent the summation of the products of the number of deaths per 100,000 inhabitants at each age, starting at age 15, and the years of life remaining up to the age ceiling, here age 65. The concept of years of life lost (YLL) was used to estimate the average years a person would have lived if he or shehad not died prematurely. YLL is a valid, stand-alone measure for identifying and ranking the causes of premature death [5,6]. It takes into account the age at which deaths occur by giving greater weight to deaths at younger age and lower weight to deaths at older age [7]. Analyses were performed with the statistical software package SAS for Windows, Version 9.2 [8].

## 3. Results

### 3.1. Overall Event Rates and Proportions

In Germany in 2010, a total of 14,441 deaths due to suicide, traffic accidents and homicide were reported. Of those 10,021 subjects (69.4%) committed suicide (7,465 men; 2,556 women), 3,942 individuals (27.3%) died in traffic accidents (2,882 men; 1,060 women) and 478 subjects (3.3%) were killed in homicides (238 men; 240 women). Event rates and proportions are given in Table 1 separately for men and women. Suicide and traffic accident mortality were higher for men than for women with respect to both rates and proportions. In both sexes, suicide rates were by far the highest, with rates for men (18.6) three times higher than for women (6.1). Traffic accident death rates came next, with rates for men (7.2) almost three times higher than for women (2.5). Homicide rates were lowest with 0.6 for both, men and women.

**Table 1:** Overall event rates (per 100,000 inhabitants) and proportions (per 100 deaths of all death causes) of suicide, traffic accidents, and homicide deaths in Germany 2010.

	Men	Women
Suicide rate	18.6	6.1
Traffic accidents rate	7.2	2.5
Homicide rate	0.6	0.6
Suicide proportion	1.8	0.6
Traffic accidents proportion	0.7	0.2
Homicide proportion	0.06	0.05

### 3.2. Age-Specific Event Rates and Proportions

Table 2 shows event rates and proportions of each unnatural death for each age group for men and women separately. For both sexes, suicide rates increased remarkably with age, whereas the proportion of suicides of all causes of death was much higher in the younger age groups. This means that elderly subjects more often committed suicide than younger persons; suicide as a cause of death, however, was considerably more frequent in younger persons.

**Table 2:** Event rates(per 100,000 inhabitants)andproportions(per 100 deaths of all deaths causes)ofsuicide (SR,SP), trafficaccident deaths (TR,TP) and homicide (HR,HP)inGermany2010,stratifiedby sexandage.

Age	SR		SP		TR		TP		HR		HP	
	m	w	m	w	m	w	m	w	m	w	m	w
**<1**	-	-	-	-	0.6	*0.6*	0.2	*0.2*	3.5	1.8	0.9	0.6
**1–4**	-	-	-	-	1.2	0.7	6.5	6.3	0.5	0.3	2.7	1.4
**5–9**	0.1	-	0.6	-	0.7	*1.7*	7.5	*9.1*	0.5	0.3	5.2	3.5
**10–14**	1.0	0.3	9.5	4.2	1.1	*1.2*	10.4	*16.0*	0.1	0.1	1.4	*1.4*
**15–19**	6.6	2.2	18.8	12.2	10.7	5.5	30.5	*30.8*	0.7	0.5	2.0	*2.9*
**20–24**	13.1	3.5	25.3	16.1	14.3	4.8	27.8	21.6	0.6	0.6	1.1	*2.8*
**25–29**	15.8	3.3	25.1	13.0	10.2	2.2	16.2	9.0	0.7	0.4	1.1	*1.6*
**30–34**	13.9	4.1	19.0	12.1	6.7	2.1	9.2	6.2	0.7	0.7	0.9	*2.2*
**35–39**	15.3	3.7	14.8	7.0	6.3	1.1	6.1	2.1	0.8	*1.2*	0.8	*2.2*
**40–44**	19.6	5.9	12.1	6.3	5.4	1.4	3.3	1.5	0.6	0.5	0.3	*0.5*
**45–49**	21.1	7.2	7.4	4.5	7.2	2.0	2.5	1.2	0.6	*0.6*	0.2	*0.4*
**50–54**	24.8	8.8	4.9	3.2	6.9	2.5	1.4	0.9	0.7	0.6	0.1	*0.2*
**55–59**	24.5	7.6	3.0	2.0	6.1	2.0	0.8	0.5	0.5	0.4	0.06	*0.1*
**60–64**	23.6	7.3	2.0	1.2	6.1	1.8	0.5	0.3	0.6	0.4	0.05	*0.1*
**65–69**	24.4	9.9	1.3	1.1	6.6	1.9	0.4	0.2	0.5	*0.5*	0.03	*0.1*
**70–74**	28.3	9.1	1.0	0.6	8.4	2.9	0.3	0.2	0.4	*0.5*	0.02	*0.03*
**75–79**	35.0	10.1	0.7	0.4	10.5	4.8	0.2	0.2	0.3	*1.1*	0.01	*0.04*
**80–84**	48.5	12.4	0.6	0.2	16.2	6.2	0.2	0.00	0.7	*0.7*	0.01	*0.01*
**85–89**	78.9	16.7	0.6	0.1	18.2	4.9	0.1	0.04	0.5	*0.8*	0.00	*0.01*
**>=90**	60.3	14.1	0.3	0.06	7.6	3.3	0.04	0.02	-	*1.2*	-	*0.01*

Equal or higher figures in the female group compared to the male group are marked by italics.

**Figure 1:**
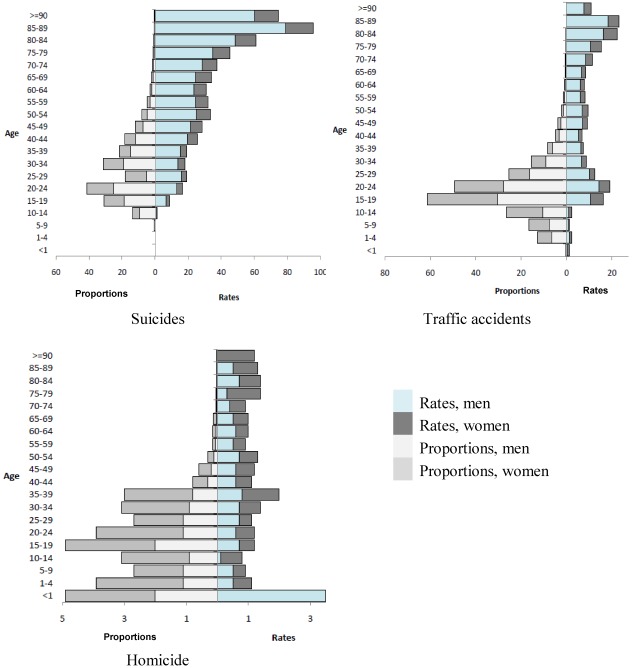
Event rates (per 100,000 inhabitants) and proportions (per 100 deaths of all causes of death) of suicide, traffic accidents, and homicide in Germany (2010), stratified by sex and age.

Different age patterns for the specific causes of unnatural death in terms of event rates (right side) and proportions (left side) are illustrated in Figure 1. Suicide deaths showed a markedly contrasting pattern between rates and proportions. For both sexes, suicide rates increased remarkably with age, whereas the proportion of suicides of all causes of death was substantially higher in younger age groups, both sexes showing a peak between 20 and 24 years.

The proportion of traffic accident deaths increased to a pronounced peak between 15 and 24 years and then steadily decreased with age, whereas traffic accident death rates exhibited a bimodal pattern with a first peak between 15 and 24 years, and a second one between 75 and 89 years.

The proportion of homicides declines steeply after the age group 35–39 years, whereas homicide rates showed a pronounced peak (dominated by men) in the age group <1 year and another peak in the age group 35–39 years. The high homicide rates in the age groups ≥70 years were dominated by women.

### 3.3. Years of Life Lost

Table 3 shows YLL due to suicide, traffic accidents and homicide per 100,000 inhabitants. In men and in women, most YLL were due to suicide, followed by traffic accidents and homicide. For men, almost twice as much YLL were due to suicide (314 YLL) than to traffic accidents (188 YLL). Furthermore, a disparity between men and women was observed, with men having 3.5 times as much YLL due to suicide than women (90 YLL) and 2.8 times as much due to traffic accidents (women: 67 YLL). Regarding homicide, both sexes showed lower years of life lost (women: 15 YLL, men 19 YLL) than for suicide or traffic accident deaths.

**Table 3:** Years of Life Lost (YLL) due to suicide, traffic accidents and homicide per 100,000 inhabitants in 2010.

	Suicide	Traffic accidents	Homicide
men	314	188	19
women	90	67	15

## 4. Discussion

The first major finding of the present study based on a population-wide data set showed that the number of deaths by suicide was 2.5 times higher than the number of deaths by traffic accidents in the year 2010 in Germany. Regarding sex-specific mortality, male predominance was distinctive in both causes of unnatural death. The two parameters event *rates* and *proportions* of mortality from unnatural death are of different reference type; therefore, they lead to different conclusions: Regarding the complex problem of suicide, increasing age was associated with a distinctive increase in *suicide rates* on the one hand – the so called Hungarian pattern [9] – and a decrease in the *proportion of suicide deaths* on the other. Hence, elderly subjects committed suicide more often than younger ones; suicide as a proportion of causes of death, however, is considerably higher in younger persons. With traffic accidents, a similar pattern in the *proportion of all death*s can be observed, whereas traffic accident death *rates* showed a u-shaped distribution indicating that the younger and the elderly more often die in traffic accidents. Remarkably, among the age group 15–19 years, the proportion of traffic accident deaths of all causes of death is much higher than the proportion of suicides. This might be explained by the fact that young individuals who newly acquired the driving license are probably more inexperienced or, due to their youth, more inclined to take unnecessary risks. The *proportion of homicides* of all causes of death stays high until an abrupt decline after 39 years of age. Homicide *rates*, on the other hand, show a rather irregular pattern. Regarding homicide rates, the high rate among infants (<1 year) is remarkable as well as the dominance of women in the very old age groups. The results of the present study blend in the general European pattern in a way that across the European Union the number for suicide deaths (55,000; rate per 100,000 inhabitants: 12.0) is higher than traffic accident deaths (48,000; rate per 100,000 inhabitants: 9.7) [10]. Furthermore, death rates for suicide are three to four times higher for men than for women across the European Union [10]. 

As for now, traffic accidents and their prevention are in the focus of interest groups and health care providers, whose efforts have resulted in steadily declining numbers of traffic deaths. Therefore, the surpassing of traffic accident deaths by suicide deaths might be a result of the rather extensive worldwide motor vehicle accident prevention from the mid 1960’s [11,12,13,14]. Suicide prevention strategies, however, must address a variety of issues: As could be shown by Baumert *et al*. (2008), different suicide methods varied in their contribution to the overall trend of suicide rates [15]. Therefore, specific methods, e.g., firearm discharge, might be main targets in suicide prevention campaigns. As the choice of suicide method was strongly related to sex and different age classes, prevention campaigns need to be designed to target specific subgroups [15,16]. 

This study could show that among suicides, traffic accidents and homicides, suicides are the main cause of death in both sexes. Rather unexpectedly, suicide is also the leading cause of YLL (men: 314 YLL, women: 90 YLL per 100,000 inhabitants). Using the concept of YLL drew specific attention to the magnitude of avoidable mortality due to suicides as YLL is a way to estimate the average years a person would have lived if he or she had not died prematurely because of committing suicide. Evidently, actions must be taken in order to establish effective means of suicide prevention.

The strengths of the current study include the use of a nationwide data base, a sampling strategy designed to limit bias in the reporting of death rates, disaggregation of the data to allow calculation of rates and proportions specific for cause of death as well as for sex and age. Additionally, in Germany, all unnatural deaths are validated by a coroner. Nevertheless, the true number of suicides might be underestimated (e.g., some of the traffic accidents might be due to suicidal intention). This potential underestimation would further underline the results of the study. Inclusion of undetermined deaths might also affect the suicide rate [15].

## 5. Conclusions

Suicide is among the leading causes of unnatural death, not only in terms of proportions and rates, but also in terms of YLL. Our results strongly indicate that within the public health care sector, suicide prevention is an issue to which priority should be given. The sex- and age-specific patterns in suicide mortality call for different action plans to specifically target high risk groups.
